# Visual Distractors Disrupt Audiovisual Integration Regardless of Stimulus Complexity

**DOI:** 10.3389/fnint.2017.00001

**Published:** 2017-01-20

**Authors:** Kyla D. Gibney, Enimielen Aligbe, Brady A. Eggleston, Sarah R. Nunes, Willa G. Kerkhoff, Cassandra L. Dean, Leslie D. Kwakye

**Affiliations:** Department of Neuroscience, Oberlin College, OberlinOH, USA

**Keywords:** multisensory integration, attention, dual task, McGurk, redundant signals effect, perceptual load, audiovisual speech, individual differences

## Abstract

The intricate relationship between multisensory integration and attention has been extensively researched in the multisensory field; however, the necessity of attention for the binding of multisensory stimuli remains contested. In the current study, we investigated whether diverting attention from well-known multisensory tasks would disrupt integration and whether the complexity of the stimulus and task modulated this interaction. A secondary objective of this study was to investigate individual differences in the interaction of attention and multisensory integration. Participants completed a simple audiovisual speeded detection task and McGurk task under various perceptual load conditions: no load (multisensory task while visual distractors present), low load (multisensory task while detecting the presence of a yellow letter in the visual distractors), and high load (multisensory task while detecting the presence of a number in the visual distractors). Consistent with prior studies, we found that increased perceptual load led to decreased reports of the McGurk illusion, thus confirming the necessity of attention for the integration of speech stimuli. Although increased perceptual load led to longer response times for all stimuli in the speeded detection task, participants responded faster on multisensory trials than unisensory trials. However, the increase in multisensory response times violated the race model for no and low perceptual load conditions only. Additionally, a geometric measure of Miller’s inequality showed a decrease in multisensory integration for the speeded detection task with increasing perceptual load. Surprisingly, we found diverging changes in multisensory integration with increasing load for participants who did not show integration for the no load condition: no changes in integration for the McGurk task with increasing load but increases in integration for the detection task. The results of this study indicate that attention plays a crucial role in multisensory integration for both highly complex and simple multisensory tasks and that attention may interact differently with multisensory processing in individuals who do not strongly integrate multisensory information.

## Introduction

We live in a world, which is rich in information from many sensory modalities. Accurately combining information from multiple senses, multisensory integration, improves our ability to function in daily life. Importantly, disruptions or alterations in multisensory integration have been associated with a number of developmental disorders (Hahn et al., 2014; Postmes et al., 2014; Wallace and Stevenson, 2014; Cascio et al., 2016). Several bottom-up features of sensory stimuli (known as the principles of multisensory integration) have been clearly demonstrated to influence the likelihood of multisensory integration: unisensory stimuli are most likely to be integrated if they share a high temporal and spatial correspondence and have low efficacy. Additionally, the principles of multisensory integration have been shown to function at the neural/cellular (Meredith et al., 1987; Meredith and Stein, 1996), network/circuit (Macaluso and Driver, 2005; Senkowski et al., 2007, 2011), and behavioral levels (Stein et al., 1988; Senkowski et al., 2011; Wallace and Stevenson, 2014). In addition to these bottom-up features, many top-down cognitive factors are known to influence or interact with multisensory integration including context (Sarmiento et al., 2016), attention (Talsma et al., 2010; Talsma, 2015; Tang et al., 2016), working memory (Quak et al., 2015), memory (Thelen et al., 2015), and emotional affect (Jessen and Kotz, 2015).

The interaction between attention and multisensory integration is the most studied of these top-down influences and has been shown to be complex and function in many directions (for review, see Talsma et al., 2010). For example, a cue in one sensory modality can capture, spatially direct, and benefit the processing of a target in another sensory modality (Driver and Spence, 1998; Pierno et al., 2005; Holmes et al., 2007; Mazza et al., 2007). In fact, multisensory objects are more effective at capturing attention than unisensory objects (Santangelo and Spence, 2007; Santangelo et al., 2008; Van der Burg et al., 2008; Spence and Santangelo, 2009). Similarly, attentional resources from one modality can spread to an unattended stimulus in another modality as long as there is a high temporal correspondence between the two stimuli (Busse et al., 2005; Molholm et al., 2007; Zimmer et al., 2010; Donohue et al., 2011).

One prominent question in the multisensory attention field is whether attention is necessary for multisensory integration. Several studies utilizing different experimental designs have demonstrated that attending to all unisensory components of a multisensory stimulus is necessary for those unisensory stimuli to be integrated. In selective attention paradigms, participants are presented with a continuous stream of auditory, visual, and audiovisual stimuli and instructed to attend to either the visual stimuli, auditory stimuli, or both the visual and auditory stimuli. Similarly, in divided attention paradigms, bimodal stimuli are presented to opposite sides of space (left and right or upper and lower hemifields), and participants are instructed to attend to only one side. These studies have consistently shown decreases in integration when participants do not fully attend both unisensory stimuli: decreased superadditive enhancement in response times in a multisensory discrimination task (Mozolic et al., 2008; Hugenschmidt et al., 2009); decreased P50 superadditivity in speeded response task (Talsma et al., 2007), decreases in event related potential (ERP) measures of the sound induced flash illusion (Mishra et al., 2010); decreased gamma band responses in a multisensory detection task (Senkowski et al., 2005).

Multisensory speech integration has been shown to be particularly sensitive to attentional demands. Morís Fernández et al. (2015) instructed participants to attend to either a high or low pitched voice to determine whether visual speech would influence accuracy for the unattended voice and found no evidence of integration for the unattended voice in both behavioral and fMRI measures. Senkowski et al. (2008) tested audiovisual speech perception of simple syllables in the presence and absence of task-irrelevant distracting flanker faces and measured steady-state visual evoked potentials (SSVEP) for both the target face and distracting faces separately. They found greater SSVEPs in response to the flanker faces in participants who showed the greatest disruption in speech identification in the presence of the flanker faces, indicating that the deployment of attention to the distracting flanker faces disrupted audiovisual speech perception (Senkowski et al., 2008). Several studies have employed a dual task design in which participants complete a primary multisensory task concurrently with a distracting task to determine whether the distracting task will decrease multisensory integration. These studies have consistently found decreases in audiovisual speech integration as indicated by decreases in reports of the McGurk illusion when attentional resources are directed to the distracting task (Tiippana et al., 2004; Alsius et al., 2005, 2007, 2014).

Although several studies have demonstrated that attention is necessary for multisensory integration, several other studies have observed that integration is unaffected by attentional manipulations. Multisensory stimuli have been shown to capture attention more effectively than unisensory stimuli and continue to be more effective even under high attentional demands. This effect has been demonstrated across several studies using a dual task experimental design and different sensory modalities including vision, audition, and somatosensation (Santangelo and Spence, 2007; Ho et al., 2009; Spence and Santangelo, 2009; Matusz et al., 2015). Single cell recordings in the superior colliculus (SC) of anesthetized cats validate that multisensory stimuli may be more effective at capturing attention in a distracting environment. Pluta et al. (2011) presented stimuli to each hemifield that were either visual, auditory, or audiovisual to determine whether the modality of a competing stimulus in the opposite hemifield would alter the neural response. They found that multisensory stimuli were more resistant to competition and were also more likely to diminish the neural response of another stimulus. Interestingly, stimulus competition led to greater superadditivity (multisensory responses greater than the sum of the unisensory responses) for multisensory stimuli in a manner similar to relatively ineffective stimuli: the overall neural responses to auditory, visual, and audiovisual stimuli were attenuated, but the neural responses to the audiovisual stimuli were relatively much greater than the sum of neural responses to the auditory and visual stimuli. This indicates that stimulus competition may actually enhance multisensory integration of neural responses in the anesthetized cat SC (Pluta et al., 2011). Several multisensory spatial tasks have also demonstrated an independence between attention and integration. Wahn and König (2015) found enhanced behavioral performance on an audiovisual (as compared to unisensory) localization task in the presence of an attentionally demanding concurrent task. Bertelson et al. (2000) found that peripheral flashes altered auditory localization (ventriloquist effect) regardless of whether attention was directed to the location of the flashes or to a central location. Similarly, when attention was exogenously directed away from the flashes, a shift in auditory localization toward the flashes was still observed (Vroomen et al., 2001a). Vroomen et al. (2001b) demonstrated that emotional multisensory stimuli were integrated under high attentional demands with their finding that a static emotional face influenced auditory emotion judgments when paired with a distracting working memory task, a visually distracting task, and an aurally distracting task.

Currently, it remains unclear what factors influence whether multisensory integration can occur in the absence of attentional resources. Several factors may influence this relationship, including which multisensory features are being integrated. For example, multisensory speech integration has been clearly demonstrated to be dependent on attention; however, multisensory spatial and emotional information may be integrated without attention (see above). One potential distinguishing feature not yet explored is whether the complexity of the multisensory stimuli or task modulates the need for attention. Multisensory integration has been demonstrated at different stages of sensory processing (Calvert and Thesen, 2004). For example, some multisensory effects such as the redundant signals effect (RSE) and sound-induced flash illusion have been localized to extrastriate visual cortex and have been shown to occur as early as 50 ms after stimulus presentation (Molholm et al., 2002; Mishra et al., 2007). However, the McGurk illusion has been localized to the superior temporal sulcus (STS) and differences in multisensory processing are not observed until 175 ms after stimulus presentation (Saint-Amour et al., 2007; Beauchamp et al., 2010). Integrative processes that occur relatively early in sensory processing may be less susceptible to top-down influences than relatively late integration (Koelewijn et al., 2010; De Meo et al., 2015). Additionally, effects of attention on multisensory integration have been observed at some, but not all, stages of processing (Ho et al., 2015). Therefore, tasks for which integration takes place at a low level of processing may be exempt from or less dependent on the need for attention than those that require a higher level of processing. Individual differences in the necessity of attention for multisensory integration have not yet been studied. Individual differences in several aspects of multisensory processing have recently been identified (Nath and Beauchamp, 2012; Stevenson et al., 2012; Mallick et al., 2015; Sun et al., 2015), which increases the likelihood that attention may interact differently with multisensory integration in some individuals. The goal of the current study was to examine whether multisensory integration in a simple task with simple stimuli would be less influenced by a competing task than a complex multisensory speech task. We also sought to test whether individuals varied in the extent to which attention altered multisensory integration.

As a measure of high complexity integration, we designed a task based on the McGurk Illusion. As reported in McGurk and MacDonald (1976) and Macdonald and Mcgurk (1978) the mouth movements for the syllable “ga” paired with the spoken auditory syllable “ba” leads to the fused perception of “da” or “tha.” Speech perception is generally considered to be highly complex and requires extensive neural processing (Cappa, 2016). Additionally, changes in speech perception under highly distracting conditions has high ecological validity due to the need to rely on mouth movements in noisy environments to improve speech intelligibility. The neural locus of integration for the McGurk illusion has been identified as the STS through a series of fMRI experiments (Baum et al., 2012; Nath and Beauchamp, 2012; Szycik et al., 2012) and a transcranial magnetic stimulation (TMS) experiment in which TMS deactivation of the STS lead to decreased reports of the McGurk illusion (Beauchamp et al., 2010). The McGurk illusion is an ideal perceptual task to measure audiovisual integration in the context of a dual task paradigm because changes in multisensory integration are indicated by changes in the percentage of fused reports that can be compared across perceptual load conditions. Lastly, several previous studies (see above) have shown decreases in the McGurk illusion under high attentional demands. In the context of this study, the McGurk task can act as a positive control to verify that our dual task design can successfully reduce multisensory speech integration.

As a measure of low complexity integration, we designed a simple speeded response or “detection” task. According to the RSE, pairings of bimodal stimuli are known to elicit faster response times than unimodal stimuli, but this difference cannot be explained by simple probability summation of the unisensory responses. The race model can be used to assess these differences by comparing the probability of a response to bimodal stimuli with the predicted summed probability to the unisensory stimuli, resulting in a measure known as Miller’s inequality (Miller, 1982; Giard and Peronnet, 1999; Molholm et al., 2002). Additionally, calculating a geometric measure of the area under the Miller’s inequality curve gives a single measure of multisensory integration that can be compared across perceptual load conditions (Colonius and Diederich, 2006; Hugenschmidt et al., 2009). Many electroencephalography (EEG) studies have found superadditive neural activity as early as 50 ms after the onset of multisensory stimuli (Giard and Peronnet, 1999; Foxe et al., 2000; Molholm et al., 2002; Murray et al., 2005). This early neural activity as well as its scalp topography strongly indicates that integration for simple speeded detection tasks occurs relatively early in sensory processing and in canonical unisensory areas (Foxe et al., 2000).

The current study seeks to determine whether directing attention away from a multisensory task in a dual task paradigm would decrease integration and whether the complexity of the multisensory task would alter the effect of attention on integration. We hypothesized that attention would be necessary for integration for the complex speech task but not for the simple speeded detection task. Thus, we predicted that increasing perceptual load would significantly decrease fused reports for the McGurk task, but would not decrease the area under the Miller’s inequality curve for the simple speeded detection task. We also capitalized on a large number of participants to study individual differences in the effects of attention on multisensory integration and how it differed across tasks.

## Materials and Methods

### Participants

A total of 109 (67 females, 18–38 years of age, mean age of 22) participants participated in this study. Eighty-six (54 females, 18–38 years of age, mean age of 22) completed the McGurk task, and 85 (54 females, 18–38 years of age, mean age of 22) completed the detection task. Sixty-two overlapped across the two tasks. Some participants completed additional experimental tasks while completing the current study procedures. Participants were excluded from final analysis if they did not complete all load conditions for at least one multisensory task or did not have a total accuracy of at least 70% on the distractor task for both load conditions. Participants reported normal to corrected-to-normal hearing and vision and no history developmental disorders or seizures. Participants gave written informed consent and were compensated for their time. Study procedures were approved by the Oberlin College Institutional Review Board and were conducted under the guidelines of Helsinki.

### Experimental Design Overview

All study procedures were completed in a dimly lit, sound-attenuated room. Participants were monitored via closed-circuit cameras for safety and to ensure on-task behavior. All visual stimuli were presented on a 24″ Asus VG 248 LCD monitor at a screen resolution of 1920 × 1080 and a refresh rate of 144 Hz that was set at a viewing distance of 50 cm from the participant. All auditory stimuli were presented from Dual LU43PB speakers which were powered by a Lepai LP-2020A+ 2-Ch digital amplifier and were located to the right and left of the participant. SuperLab 4.5 software was used for stimulus presentation and participant response collection. Participants indicated their responses on a Cedrus RB-834 response box, and responses were saved to a txt file.

This study employed a dual task design to determine whether distracting attention from a multisensory task would alter the degree of integration and whether this effect depended on the level of complexity of the multisensory stimuli being integrated. Similar dual task designs have been shown to reduce attentional capacity for the secondary task (Lavie et al., 2003; Stolte et al., 2014; Bonato et al., 2015). Participants completed a multisensory task concurrently with a distractor task to address the research question. A task based on the McGurk illusion was used to test high complexity multisensory integration, and an audiovisual speeded detection task was used to test low complexity multisensory integration. Perceptual load was varied for the distractor task to titrate the attentional resources distracted from the multisensory tasks. All study procedures related to each multisensory task were completed together, and the order of completion of the multisensory tasks was randomized across participants. Participants completed each multisensory task at varying perceptual loads of the distractor task, and each load condition was separated into blocks. Further, the order of the load condition blocks was randomized across participants. Thus each block tested a particular multisensory task by perceptual load condition and were randomized first by multisensory task then by perceptual load. For each block, participants first practiced the multisensory task without any distracting stimuli. They then practiced the multisensory task with the additional instructions for that perceptual load.

### Distractor Task

Stimuli consisted of rapid serial visual presentations (RSVP) of white and yellow letters and white numbers subtending a 3.5° visual angle and presented 8.5° below center for the McGurk task and at center for the speeded detection task. Some letters (I, B, O) and numbers (1, 8, 0) did not appear in the RSVP streams because the visual similarity between the letters and numbers would be confusing for participants. The RSVP stream was presented continuously during the presentation of the McGurk videos and before and after the speeded detection stimuli (see below). Each letter/number in the RSVP stream was presented for 100 ms with 20 ms between letters/numbers. The distractor task included three condition types: no perceptual load (NL), low perceptual load (LL), and high perceptual load (HL). The participant was presented with an RSVP stream and either asked to ignore it (NL), detect infrequent yellow letters (LL), or detect infrequent white numbers (HL). Previously published dual task studies have utilized similar RSVP streams composed of letters and numbers with a color change representing a low load target and/or a number representing a high load target (Santangelo and Spence, 2007; Santangelo et al., 2008; Parks et al., 2011; Asanowicz et al., 2013). Each RSVP stream had a 25% probability of containing no numbers or yellow letters, a yellow letter only, a number only, or a yellow letter and number resulting in a 50% probability of a target being present for the LL and HL conditions. After each trial, participants were asked to respond first to the multisensory task then report with a “yes” or “no” button press whether they observed a target for that trial. Each load condition was completed in a separate block, and participants were able to take breaks between blocks. The order that participants completed the load condition blocks was randomized and counterbalanced across participants.

### Experiment 1: McGurk

**FIGURE 1:**
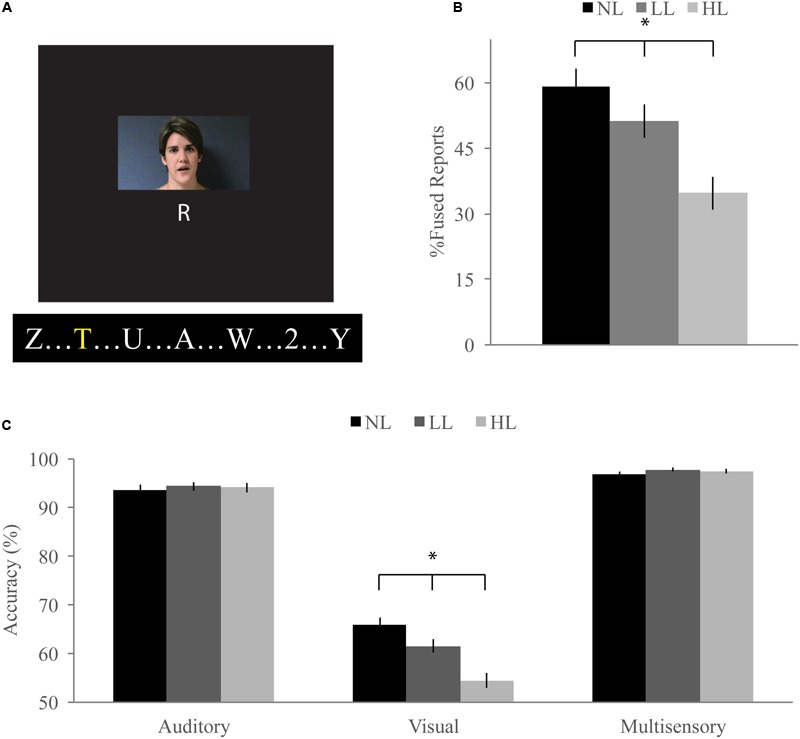
**McGurk task. (A)** Participants watched short movies of a woman speaking and reported what syllable they perceived at the end of each trial while viewing a stream of letters and either ignoring them [no load (NL)], reporting infrequent yellow letters [low load (LL)], or reporting infrequent numbers [high load (HL)]. Written informed consent was obtained for the publication of this identifiable image. **(B)** Percent fused reports for illusory incongruent trials across perceptual load. Fused reports significantly decreased with increasing perceptual load. **(C)** Accuracy for visual, auditory, and congruent multisensory trials. Accuracy for visual trials significantly decreased with increasing load. Error bars represent the standard error of the mean (SEM). ^∗^Indicate significant (*p* < 0.05) differences across loads.

Videos for the McGurk task were made using Ulead VideoStudio 11 with a frame rate of 30 fps and lasted 700 ms. Videos subtended 21.5° horizontally and 12.5° vertically and were presented at 65 dB SPL (**Figure 1A**) For each trial type in the McGurk task, a video of a woman speaking a syllable (“ba,” “da,” “ga,” or “tha”) was presented at the center of the screen after an initial 500 ms prestimulus interval. After the offset of the video, a response slide was presented which asked “what did she say?” Participants were instructed to respond by pressing a button on the response box for one of the following options: “ba,” “da,” “ga,” or “tha.”

There were five total trial types, and the order of trial types was randomized at the beginning of the block. Unisensory trials ensured that participants attended to both the auditory and visual components of the videos. During visual trials, a silent video of a woman speaking one of the syllables (“ba,” “da,” “ga,” or “tha”) was played. Each syllable was presented eight times per load condition for a total of 32 visual only trials per block. During the auditory trials, a still image of the woman’s face was presented along with the audio of her speaking one of the syllables (“ba,” “da,” “ga,” or “tha”). Each syllable was presented eight times per load condition for a total of 32 auditory only trials per block. During multisensory congruent trials, unaltered videos of a woman speaking one of the syllables (“ba,” “da,” “ga,” or “tha”) was presented such that the mouth movements of the woman matched the spoken syllable. Each syllable was presented eight times per load condition for a total of 32 multisensory congruent trials block. During multisensory incongruent trials, videos were presented for which the mouth movements of the woman speaking did not match the spoken syllable. There were two types of multisensory incongruent trials: illusory and non-illusory. Illusory trial videos were composed of a visual “ga” and an auditory “ba” which has been shown to produce the McGurk Illusion (McGurk and MacDonald, 1976). Non-illusory trials were composed of a visual “ba” and an auditory “ga.” Illusory and non-illusory trials were repeated 16 times each per load condition for a total of 32 incongruent trials per block.

### Experiment 2: Speeded Detection Task

**FIGURE 2:**
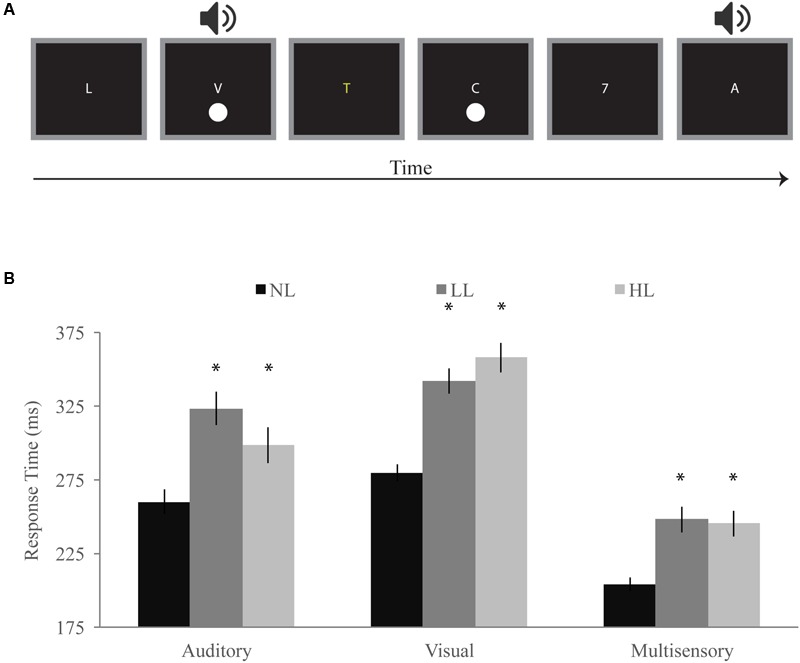
**Detection task. (A)** Participants responded with a speeded button press when they detected a visual, auditory, or simultaneous audiovisual stimulus while viewing a stream of letters and either ignoring them (NL), reporting infrequent yellow letters (LL), or reporting infrequent numbers (HL). **(B)** Response times for visual, auditory, and multisensory trials for NL, LL, and HL blocks. Multisensory response times were significantly faster for each perceptual load, and response times generally increased with increasing load. Error bars represent the SEM. ^∗^Indicate significant (*p* < 0.05) differences from NL.

There were three trial types for the speeded detection task: visual only (60 ms circle flash), auditory only (60 ms white noise burst), and multisensory (simultaneous 60 ms circle and white noise burst), the order of which was randomized (**Figure 2A**). Each trial type was repeated 30 times per load for a total of 90 trials per block. Visual stimuli for the speeded detection task consisted of a white circle that subtended a visual angle of 4.5° and was presented for 60 ms at an eccentricity of 8.5° below the central fixation. Auditory stimuli consisted of a centrally presented white noise burst at 60 dB SPL for 60 ms. For each trial type for the speeded detection task, visual and/or auditory stimuli were presented after an initial variable pre-stimulus interval, which ranged in duration from 500 to 1400 ms. After the presentation of the auditory and/or visual stimulus there was a poststimulus interval of 1780 ms during which the participant would indicate via button press that they detected the visual and/or auditory stimulus. Participants were asked to respond as quickly as possible. The RSVP stream was presented in the center of the screen continuously throughout the trials including the initial variable prestimulus interval and the poststimulus interval. Upon the termination of the poststimulus interval in the LL and HL conditions, a response screen would appear asking the participant to indicate the presence of a target (yellow letter for LL or number for HL). Upon the termination of the poststimulus interval in the NL condition, the next trial would begin without asking the participant for a response.

### Data Analysis

#### McGurk

Responses on incongruent trials were divided into visual reports (participant reported the syllable that matched the lip movements of the speaker), auditory reports (participant reported the syllable that matched the spoken syllable), and fused reports (“da” or “tha”). The percent auditory, visual, and fused reports were calculated for each participant separately for each load condition and incongruent trial type (illusory versus non-illusory). To determine whether we successfully elicited the McGurk illusion and whether perceptual load influenced multisensory integration for this task, we conducted a repeated-measures analysis of variance (RMANOVA) with percent fused reports as the dependent factor and condition (illusory or non-illusory) and load (NL, LL, or HL) as independent factors. We then conducted planned paired-samples *t*-tests for fused reports between illusory and non-illusory trials for each load condition to confirm whether the McGurk illusion was elicited for each load condition. Lastly, we compared fused reports between each load condition to determine if an increase in load significantly changed multisensory integration on the McGurk task.

Unisensory and congruent multisensory trials were included to determine whether increasing perceptual load affected speech perception generally and whether the modality of the speech influenced this interaction. Accuracy on auditory, visual, and multisensory congruent trials was calculated within perceptual load and averaged across syllables creating a single auditory, visual, and multisensory congruent percent accuracy for each load condition. We conducted a RMANOVA with accuracy as a dependent factor and modality (auditory, visual, or multisensory) and perceptual load (NL, LL, or HL) as independent factors to determine whether perceptual load influenced speech perception. We then conducted planned paired-sample *t*-tests on accuracy for each modality between load conditions to determine whether perceptual load altered accuracy for each modality.

We calculated a dual task effect (DTE) for changes in accuracy and fused reports between HL and NL (but not between NL and LL) to compare across trial types and multisensory task types. The DTE represents the percent change in performance for HL as compared to NL and was calculated as:

(1) DTE=[(HL−NL)/NL]×100

Similar measures have been used in other published dual task paradigm studies (Plummer and Eskes, 2015). Participants were excluded from this analysis and classified as non-integrators if a DTE score would be incalculable because they had zero percent fused reports for the NL block (14 participants). Because a change in unisensory speech intelligibility could alter fused reports independent of changes in multisensory integration, we correlated DTEs for fused reports with DTEs for accuracy for each modality (visual, auditory) separately using Pearson’s correlation.

#### Speeded Detection

Response times occurring between 100 and 1100 ms were averaged within load condition and separately based on modality (auditory, visual, audiovisual). A RMANOVA was conducted with average response time as a dependent factor and load and modality as independent factors. Miller’s Inequality is a well-accepted and often used measure of multisensory integration for this task (Molholm et al., 2002, 2006; Colonius and Diederich, 2004; Diederich and Colonius, 2004) and was used as a measure to assess whether participants integrated the audiovisual stimuli for each load and whether integration changed as a function of load. Response times for each trial type were first binned into 20 equal 5% quantiles, and a cumulative probability was calculated for each quantile resulting in a cumulative probability function (CPF). The race model CPF was then calculated by summing the CPFs of the auditory and visual responses for each quantile up to a maximum probability of 1:

(2) $$CPF_{\text{RM}}=CPF_{\text{V}}+CPF_{\text{A}}$$

Miller’s Inequality was calculated for each load condition by subtracting the race model CPF from the multisensory CPF. Paired sample *t*-tests were conducted between the race model CPF and multisensory CPF for the first five quantiles for each load to determine which quantiles showed significantly faster response times for multisensory stimuli as compared to the predictive sum of unisensory responses. Additionally, quantiles for Miller’s inequality were compared between NL and HL to determine whether integration differed with increasing load. Alpha error was controlled by limiting the number of quantiles compared and by Bonferroni correcting the alpha level to *p* = 0.0025. We then calculated a geometric measure of Miller’s inequality for each participant to generate a single measure of multisensory integration to compare across loads. We specifically calculated the positive area under the Miller’s inequality curve (pAUC) because of its use in previous studies as a measure of integration and its interpretability (Nozawa et al., 1994; Hughes et al., 1998; Colonius and Diederich, 2006; Hugenschmidt et al., 2009; Stevenson et al., 2014). We determined the pAUC by calculating the trapezoidal area between each quantile that produced a positive Miller’s inequality. Each trapezoidal area was summed to give a total pAUC for each load condition. We then compared the resulting pAUC across load conditions using a RMANOVA and paired samples *t*-tests.

We calculated a DTE for changes in response time and pAUC between HL and NL to compare across trial types and multisensory task types. The sign of the DTE scores for response time were reversed so that detriments in performance (greater response times) would be indicated by a negative DTE score. Participants were excluded from this analysis if a DTE score would be incalculable because they had zero pAUC for the NL block (16 participants). We correlated DTEs for pAUC with DTEs for response time for each modality (visual, auditory) separately using Pearson’s correlation.

#### Comparisons Across Tasks

We compared changes in multisensory integration with increasing perceptual load across the McGurk and speeded detection tasks. We calculated DTEs for fused reports as a measure of changes in integration for the McGurk task and DTEs for the pAUC as a measure of changes in integration for the speeded detection task. We then compared the DTEs using an independent samples *t*-test. To determine whether changes in integration were associated across tasks, we correlated the DTEs across tasks using Pearson’s correlation.

To determine how multisensory integration changes with increasing perceptual load for non-integrators, we compared changes in fused reports for the McGurk task and changes in pAUC for the detection task across loads using paired samples *t*-tests.

#### Distractor Task Performance

Percent accuracy for identifying targets on the distractor task was calculated for each multisensory task and separated by the modality of the trial type. A RMANOVA was conducted with accuracy as the dependent factor and task (McGurk or Detection), load (LL or HL) and modality (visual, auditory, or multisensory) as independent factors. A significant main effect of load would confirm the overall increase in difficulty (and the presumed increased allocation of attention) for the HL distractor task. Significant main effects of or interactions with modality would indicate that participants actively altered their attention allocation from trial to trial depending on the modality of the stimulus. A significant main effect of task would indicate that the amount of attention directed to the distractor task may differ between multisensory tasks.

## Results

Participants completed a dual task paradigm that included a multisensory task of low or high complexity (speeded detection or McGurk, respectively) and a distractor task that varied in perceptual load (NL, LL, HL). These tasks were used to determine whether directing attention away from a multisensory task would decrease multisensory integration on that task and whether the complexity of the multisensory task modulated this interaction.

### Experiment 1: McGurk Task

Participants viewed videos of a woman speaking simple syllables and were asked to report which syllable they perceived (“ba,” “da,” “ga,” or “tha”). Trial types consisted of visual (silent videos), auditory (still image of the woman’s face with the auditory syllable), congruent multisensory (unaltered video of each syllable), and incongruent multisensory. The incongruent multisensory trials were further subdivided into illusory (visual “ga” and auditory “ba”) and non-illusory (visual “ba” and auditory “ga”). Unisensory and congruent multisensory responses were coded for response accuracy. Incongruent responses were coded as being auditory (matching the auditory syllable in the video), visual (matching the visual syllable in the video), or fused (not present in the video: “da” or “tha”).

#### Incongruent Trials

Our McGurk task successfully elicited the McGurk illusion as evidenced by significantly greater fused reports for illusory incongruent trials (visual “ga” auditory “ba”) as compared to non-illusory incongruent trials (visual “ba” auditory “ga”) for NL [59% fused reports for illusory, 2% fused reports for non-illusory; *t*(85) = 16.41, *p* < 0.001], LL [51% fused reports for illusory, 2% fused reports for non-illusory; *t*(85) = 9.09, *p* < 0.001], and HL [35% fused reports for illusory, 2% fused reports for non-illusory; *t*(85) = 11.32, *p* < 0.001] blocks (**Figure 1B**). To determine whether increasing perceptual load influenced integration on the McGurk task, we conducted a RMANOVA on fused reports with condition (illusory versus non-illusory) and perceptual load (NL, LL, HL) as factors. The main effect of condition was significant [*F*(1,85) = 194.04, *p* < 0.001] confirming that participants reported fused percepts more often for illusory than non-illusory trials. The main effect of load [*F*(2,170) = 30.83, *p* < 0.001] and the interaction between load and condition [*F*(2,170) = 30.43, *p* < 0.001] were significant, indicating that perceptual load influenced fused reports and that this effect was dependent on whether the stimuli were illusory or non-illusory. Direct comparison paired-sample *t*-tests showed that fused reports significantly decreased for illusory trials between NL and LL [mean difference of 2.80, *t*(85) = 2.55, *p* = 0.012], NL and HL [mean difference of 24, *t*(85) = 7.66, *p* < 0.001], and LL and HL [mean difference of 22, *t*(85) = 5.29, *p* < 0.001]. Importantly, fused reports did not significantly differ between any load conditions for non-illusory trials.

#### Unisensory and Congruent Trials

Unisensory and congruent multisensory trials were used to determine whether increasing perceptual load affected speech perception generally and whether the modality of the speech influenced this interaction (**Figure 1C**). A RMANOVA with response accuracy as the dependent factor and modality and perceptual load as independent factors revealed that both modality [*F*(2,168) = 799.21, *p* < 0.001] and perceptual load [*F*(2,168) = 11.90, *p* < 0.001] significantly influenced response accuracy. The effect of perceptual load on response accuracy was modulated by the modality of the syllable as evidenced by a significant modality by load interaction [*F*(4,336) = 20.585, *p* < 0.001]. Response accuracy for visual syllables (NL: 66%, LL: 62%, HL: 54%) significantly decreased as perceptual load increased (NL/LL *t*(85) = 3.19, *p* = 0.002; NL/HL *t*(85) = 6.95, *p* < 0.001; LL/HL *t*(85) = 4.25, *p* < 0.001). However, response accuracy for auditory (NL, LL, HL: 94%) and congruent multisensory (NL: 97%, LL: 98%, HL: 98%) syllables did not significantly differ across perceptual load conditions [Auditory: NL/LL *t*(85) = -0.40, *p* = 0.689; NL/HL *t*(85) = -0.32, *p* = 0.754; LL/HL *t*(85) = 0.11, *p* = 0.909; Congruent Multisensory: NL/LL *t*(85) = -1.55, *p* = 0.126; NL/HL *t*(85) = -1.00, *p* = 0.321; LL/HL *t*(85) = 0.59, *p* = 0.560]. Taken together, these results show that increasing visual perceptual load specifically disrupts syllable identification for visual stimuli only. DTEs for visual and auditory accuracy were individually correlated with DTEs for fused reports (**Figure 4A**). Changes in accuracy did not significantly predict changes in fused reports for either modality [Visual: *r*(72) = 0.151, *p* = 0.206, Auditory: *r*(72) = 0.099, *p* = 0.407] indicating that changes in unisensory accuracy are not associated with changes in fused reports.

### Speeded Detection Task

Participants made speeded responses to auditory, visual, and audiovisual stimuli using a response box. Response times were averaged within stimulus type to determine whether the RSE was observed in each load condition. CPF were also calculated to determine whether audiovisual responses violated the race model for each load condition.

#### Overall Response Times and the Redundant Signals Effect

Response times for visual, auditory, and audiovisual trials were averaged within each load condition and a RMANOVA was conducted with load and modality as independent factors (**Figure 2B**). Modality significantly influenced response times [*F*(2,168) = 234.07, *p* < 0.001] as did perceptual load [*F*(2,168) = 49.10, *p* < 0.001]. Interestingly, we found a significant interaction between modality and load [*F*(4,336) = 16.30, *p* < 0.001] indicating that the effect of load on response time differs across modalities. Response times for audiovisual trials were significantly faster than both auditory [NL: *t*(84) = 10.58, *p* < 0.001; LL: *t*(84) = 12.00, *p* < 0.001; HL: *t*(84) = 9.07, *p* < 0.001] and visual [NL: *t*(84) = 22.67, *p* < 0.001; LL: *t*(84) = 20.22, *p* < 0.001; HL: *t*(84) = 21.25, *p* < 0.001] response times for each load condition. Visual response times (NL: 280 ms; LL: 342 ms; HL: 358 ms) significantly increased with increasing load [NL/LL: *t*(84) = 9.77, *p* < 0.001; NL/HL: *t*(84) = 10.63, *p* < 0.001; LL/HL: *t*(84) = 2.28, *p* = 0.025]. Auditory response times (NL: 260 ms; LL: 323 ms; HL: 298 ms) significantly increased between NL and LL [*t*(84) = 7.43, *p* < 0.001] blocks but decreased between LL and HL blocks [*t*(84) = -2.87, *p* = 0.005]. However, auditory response times were significantly longer for HL as compared to NL [*t*(84) = 4.58, *p* < 0.001]. Response times for audiovisual trials [NL: 204 ms; LL: 248 ms; HL: 245 ms] were significantly higher for LL [*t*(84) = 6.23, *p* < 0.001] and HL [*t*(84) = 6.07, *p* < 0.001] as compared to NL but did not significantly differ between LL and HL blocks [LL/HL: *t*(84) = 0.53, *p* = 0.601].

#### Cumulative Probability Functions and Tests of the Race Model

**FIGURE 3:**
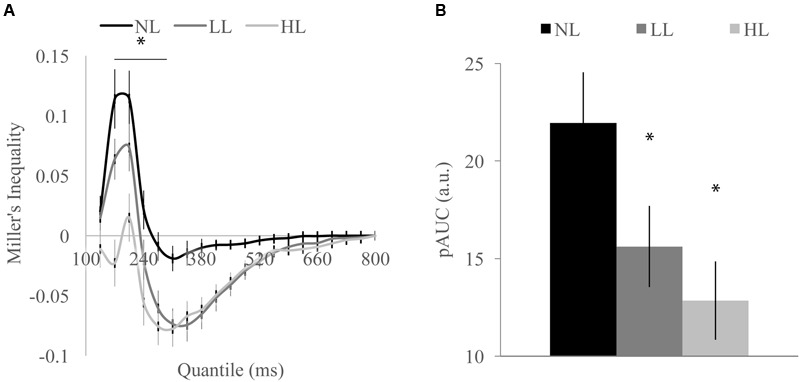
**Miller’s inequality across perceptual load conditions for detection task shown by quantile (A)** and total positive area under the Miller’s inequality curve (AUC) **(B)**. Miller’s inequality decreased with increasing load. Error bars represent the SEM. ^∗^Indicate contiguous significant (*p* < 0.001) differences between HL and NL **(A)**. ^∗^Indicate significant (*p* < 0.001) differences from NL **(B)**.

**FIGURE 4:**
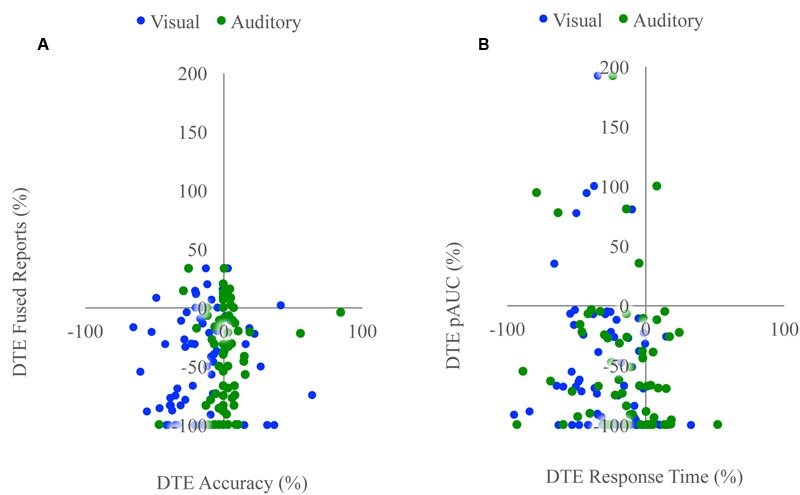
**Associations with changes in unisensory performance.** Scatterplots showing McGurk task fused reports DTE with accuracy DTE **(A)** and detection task positive area under the Miller’s inequality curve DTE with response time DTE **(B)**. The sign of the DTE for response time has been reversed so that decreases in performance (increased response time) would be represented as a negative DTE. Changes in unisensory performance were not associated with changes in multisensory integration for either task.

We tested whether the RSE observed for each load condition exceeded the facilitation predicted by probability summation using Miller’s inequality. We first calculated CPFs of the race model for each load condition and then subtracted the CP predicted by the race model from the observed CP in response to audiovisual stimuli at each quantile to determine the Miller’s inequality (Supplementary Figure 1). We found significantly positive Miller’s inequalities (observed audiovisual CP greater than race model prediction) in the NL condition for the 170 [*t*(84) = 4.62, *p* < 0.001] and 205 [*t*(84) = 4.93, *p* < 0.001] ms quantiles, in the LL condition for the 170 [*t*(84) = 3.75, *p* < 0.001] and 205 [*t*(84) = 3.73, *p* < 0.001] ms quantiles, and in no quantiles for the HL condition (**Figure 3A**). Miller’s inequality was significantly greater in the NL condition as compared to the HL condition for the 135 [*t*(84) = 2.28, *p* = 0.025], 170 [*t*(84) = 6.91, *p* < 0.001], 205 [*t*(84) = 4.53, *p* < 0.001], 240 [*t*(84) = 3.90, *p* < 0.001], and 275 ms quantiles [*t*(84) = 3.86, *p* < 0.001]. We calculated the pAUC (NL = 21.9, LL = 15.6, HL = 12.8; **Figure 3B**). A RMANOVA revealed that load significantly modulated the pAUC [*F*(2,168) = 16.18, *p* < 0.001]. Planned paired-samples *t*-test demonstrated that the decrease in the pAUC specifically occurred between NL and LL blocks [*t*(84) = 3.90, *p* < 0.001] but approached significance between LL and HL blocks [*t*(84) = 1.96, *p* = 0.053]. However, the area under the Miller’s inequality curve was significantly greater in the NL as compared to the HL blocks [*t*(84) = 4.91, *p* < 0.001]. Similar to the McGurk task, changes in unisensory performance (DTEs for response time) did not significantly predict changes in multisensory integration (DTEs for Miller’s inequality area under the curve) on the speeded detection task [visual: *r*(68) = 0.008, *p* = 0.951; auditory: *r*(68) = -0.124, *p* = 0.311] (**Figure 4B**).

### Associations between McGurk and Speeded Detection Tasks

**FIGURE 5:**
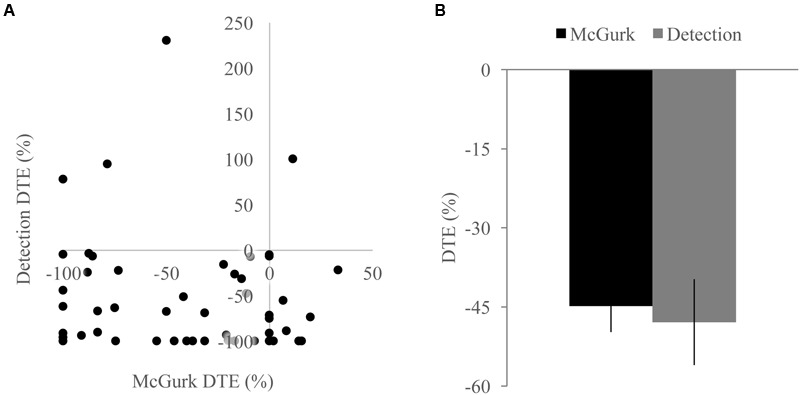
**Differences across tasks. (A)** Scatterplot showing McGurk fused reports dual task effect (DTE) and detection positive area under the Miller’s inequality curve DTE. **(B)** Average DTE across tasks. Decreases in multisensory integration were not significantly different or correlated between the two tasks. Error bars represent the SEM.

**FIGURE 6:**
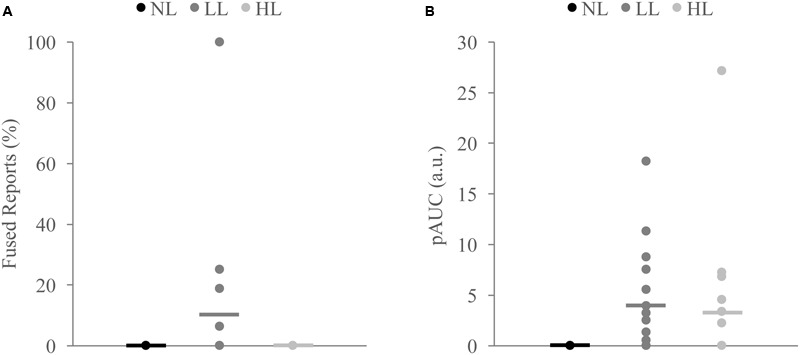
**Measures of integration across load for non-integrators on the McGurk (A)** and detection **(B)** tasks. Non-integrators on the McGurk task did not significantly report the McGurk illusion for any perceptual load condition **(A)**. Non-integrators on the detection task showed significant increases in the positive area under the Miller’s inequality curve (AUC) between NL and LL **(B)**. Horizontal lines represent the mean for each load. Indicate significant differences from NL.

Because we found decreases in measures of multisensory integration with increasing perceptual load for both the McGurk and speeded detection tasks, we directly compared the DTEs for fused reports to DTEs for pAUC to determine whether these changes were equivalent and associated across tasks (**Figure 5**). DTEs for fused reports and pAUC were not significantly different [*t*(139) = 0.32, *p* = 0.748], nor were they significantly correlated [*r*(52) = -0.085, *p* = 0.544] indicating that decreases in multisensory integration were similar but independent across tasks. Importantly, participants who were excluded from the DTE analysis because they did not show evidence of multisensory integration on the NL tasks had a different pattern of changes in integration across loads (**Figure 6**). Participants excluded from the McGurk showed no evidence of integration (fused reports significantly greater than 0) for any load; however, participants excluded from the speeded detection task showed greater pAUC for the LL block as compared to the NL block [*t*(15) = 3.00, *p* = 0.009].

### Distractor Task Performance

**FIGURE 7:**
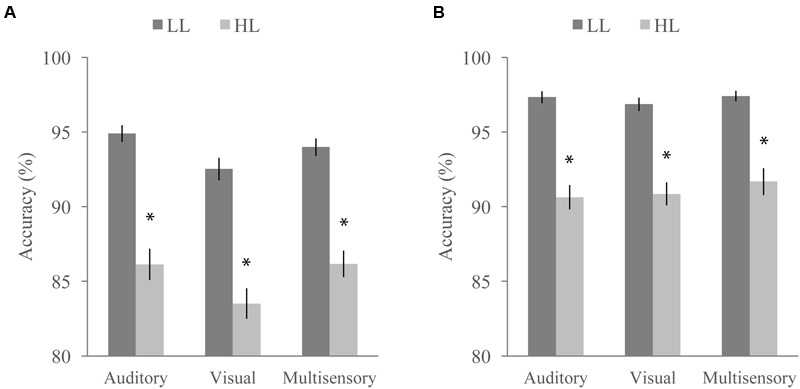
**Average accuracy for the distractor task when paired with the McGurk (A)** and detection **(B)** tasks. Accuracy significantly differed as a function of task, perceptual load, and stimulus modality. Error bars represent the SEM. ^∗^Indicate significant differences (*p* < 0.05) between LL and HL.

Concurrent with the multisensory tasks, participants viewed a RSVP stream of letters and numbers and were asked to ignore them (NL), report the presence of a yellow letter (LL), or report the presence of a number (HL) at the end of each trial. Targets were present in 50% of the trials. We conducted a RMANOVA with response accuracy on the RSVP distractor task as the dependent variable and perceptual load (LL or HL), multisensory task (McGurk or speeded detection), and modality of the multisensory task stimulus (auditory, visual, or audiovisual) as factors (**Figure 7**). Response accuracy was significantly influenced by perceptual load [*F*(1,64) = 201.91, *p* < 0.001] and was higher for LL than HL for both tasks (overall mean accuracy of 95.83 for LL and 88.43 for HL). The accompanying multisensory task also significantly influenced response accuracy on the distractor task [*F*(1,64) = 45.50, *p* < 0.001] with the speeded detection task leading to greater accuracy (overall mean accuracy of 94.33 for the speeded detection task and 89.93 for the McGurk task). We also found a significant main effect of modality [*F*(2,128) = 7.39, *p* = 0.001], indicating that the stimulus modality on the multisensory tasks influenced accuracy on the concurrent RSVP distractor task. A significant interaction between multisensory task and load [*F*(1,64) = 6.34, *p* = 0.014] indicates that the difference in accuracy between HL and LL conditions depends on which multisensory task the distractor task was paired with. We also found a significant interaction between task and modality indicating that the modality of the stimulus had a different effect on accuracy depending on the multisensory task [*F*(2,128) = 3.90, *p* = 0.023]. The interaction between modality and load [*F*(2,128) = 1.04, *p* = 0.354] as well as the three-way interaction between modality, load, and task [*F*(2,128) = 0.71, *p* = 0.494] were not significant.

## Discussion

### Conclusion

We report consistent decreases in multisensory integration for two well-established but very different multisensory tasks with increased attentional demands. These decreases in multisensory integration are evidenced by decreases in the McGurk illusion and decreased pAUC, which indicates less multisensory facilitation in response times with increased difficulty on the corresponding distractor task. Our data are consistent with previous findings of decreases in multisensory speech integration under high attentional demands, strongly suggesting that attention is necessary for high-complexity integration of audiovisual speech. However, our results from the detection task indicate that attention is also likely necessary for multisensory integration that occurs early in stimulus processing. These observed decreases in integration in addition to previously reported findings strongly suggest that attention is necessary for multisensory integration in the vast majority of circumstances and that attention may be unnecessary in only a few exceptional cases. Some studies have suggested that multisensory integration may occur regardless of the effect of attention (Vroomen et al., 2001b; Ho et al., 2009), and that perceptual load does not influence integration (Santangelo and Spence, 2007). Thus, there is some variance regarding the necessity of attention for multisensory integration. The current study presents evidence that neither stimulus nor task complexity modulate this difference, although it is possible that integration that occurs very early in the stimulus processing hierarchy (possibly subcortical) is less dependent on attention but that our detection task is actually still too complex to measure such low-level integration. Additionally, because this study utilized a dual-task paradigm which reduces the perceptual capacity available to process multisensory information, interactions between multisensory integration and other manipulations of attention may not follow the same pattern. However, previous studies have utilized a variety of methods to manipulate attention in multisensory tasks, and the method used does not seem to predict whether attention is necessary for multisensory integration. We also found changes in unisensory performance for both tasks as evidenced by decreases in visual speech accuracy in the McGurk task and increased response times in the detection task. However, changes in unisensory performance did not predict changes in integration for either task.

Our study design allowed us to directly compare changes in multisensory integration with increasing attentional demands for both a high complexity speech task and low complexity detection task to determine whether a reduced perceptual capacity would alter integration similarly for both tasks. We found that decreases in multisensory integration with increasing load were roughly equivalent across tasks. Although both stimulus and task complexity were manipulated simultaneously, our results indicate that neither stimulus nor task complexity alter the effect of attention of multisensory integration. Additionally, changes in integration with increasing load were not associated between tasks. Taken together, these results indicate that attention is equally necessary for both high and low complexity multisensory integration but that it interacts differently with the integrative mechanisms for both tasks. This may suggest that the neural mechanisms of attention on the two tasks may be different or possibly reflect different neural mechanisms of integration that automatically interact differently with attention.

Our study offers novel insight into individual differences in the effects of attention on multisensory integration. Interestingly, there were participants who showed increases in integration with increasing load indicating that attention may be unnecessary for integration for some individuals. We also observed differences between tasks for participants who did not show evidence of multisensory integration for the NL block. Participants who did not perceive the McGurk illusion for the NL block continued not perceiving the illusion for both the LL and HL blocks, indicating that attention did not alter their lack of multisensory integration for this speech task. However, some participants whose response times to multisensory stimuli did not violate the race model for the NL block actually showed superadditive response times with increasing load. Although these participants represent a small minority, our finding of increases in integration with increasing load on the detection task but no changes in integration on the McGurk task represents a key difference in the effects of attention on multisensory integration for these two tasks.

We also noted a number of differences in performance on the distractor task depending on which multisensory task the distractor task was paired with and even the sensory modality of the stimuli on the accompanying multisensory task. Unsurprisingly, we found differences in accuracy between load levels: high load was harder, confirming that more attentional resources were necessary for it. Overall, participants performed better on the distractor task when it was paired with the speeded detection task as opposed to the McGurk task. This suggests that participants are optimizing their performance and directing more attention to the distractor task when it is paired with the easier detection task. Interestingly, we found changes in performance on the distractor task as a function of the stimulus modality for the multisensory task. This suggests that participants are actively directing their attention on a trial by trial basis to optimize their performance. This fluid allocation of attention creates a potential limitation for the study results since we cannot precisely control the “amount” of attention directed to the multisensory tasks. However, our study is likely capturing interactions between multisensory integration and attention that are much more representative of realistic functioning. Future neuroimaging studies could help to elucidate how attention is flexibly directed to a multisensory task in a multitasking/dual-task environment.

### Potential Neural Mechanisms

There are a number of neural mechanisms that may underlie attentional influences on multisensory integration. One potential mechanism for how a reduced perceptual capacity may disrupt multisensory integration is through interference with higher order processes that modulate integration. For example, the anterior ectosylvian sulcus (AES) in the anesthetized cat was shown to modulate integration in the SC through a pivotal cortical deactivation experiment. Prior to and after deactivation of the AES, multisensory responses in SC were predominantly superadditive; however, when AES was deactivated, multisensory responses in SC neurons switched to being primarily additive. This study demonstrated that SC neurons often receive inputs from multiple modalities, but cannot actively enhance the response to a multisensory stimulus without top-down cortical input (Jiang et al., 2001). Top-down involvement in cortical multisensory integration has also been demonstrated for speeded detection tasks. Brang et al. (2013) found an association between increased connectivity between parietal cortex and early sensory areas with increased response time facilitation for audiovisual targets suggesting that increased connectivity between early sensory areas and parietal cortex may mediate superadditive response times in multisensory detection tasks. In the context of the present study, decreased facilitation for audiovisual targets with increasing perceptual load may have occurred due to disruptions in parietal functioning because of the attentional demands of the distracting task. Future studies measuring changes in functional connectivity between early sensory areas and parietal cortex with increasing perceptual load could determine the importance of this mechanism for the effects of attention on multisensory integration.

Another potential neural mechanism is that a reduced perceptual capacity may disrupt the processing of the unisensory signal independent of multisensory integration such that degraded unisensory signals are integrated. The effect that this would have on integration would vary depending on the multisensory task. For example in the McGurk task, one would expect an overall decrease in integration. Attention has been shown to improve the neural encoding of auditory speech in lower-order areas and to selectively encode attended speech in higher order areas (Zion Golumbic et al., 2013). In the context of the McGurk task, a disrupted neural representation of visual speech may have less of an influence on auditory speech perception. We report decreases in visual accuracy and decreases in fused reports with increasing load, which supports the hypothesis that disruptions of the visual processing of speech may underlie the decreases in integration for the McGurk task. An auditory distractor task may have less of an influence on integration or even heighten the effect of visual speech with increasing auditory perceptual demands. An experiment which utilizes both visual and auditory distractors and models changes in integration using Bayesian Causal Modeling may be able to identify changes in unisensory reliability with increasing perceptual load for each modality. Similarly, neuroimaging studies may be able to isolate unisensory-specific processing and show whether changes in unisensory processing occur with changes in attentional demands.

Attention may also help promote integration by inducing oscillatory activity in unisensory and multisensory areas and by helping to synchronize the activity between these areas. Several studies have demonstrated that multisensory integration is dependent on oscillatory activity in the gamma frequency range (Senkowski et al., 2005; Mishra et al., 2007). Attentional resources may be necessary to instigate oscillations and to synchronize them across brain areas. Attention and oscillatory synchrony have also been shown to interact in a number of studies (Gomez-Ramirez et al., 2011; Keil et al., 2016), thus strengthening the possibility of this potential mechanism. The speeded response task has been associated with phase resetting in a study by Mercier et al. (2015). They found that visual stimuli reset the phase of ongoing auditory processing and thus modulated auditory activity. Additionally, greater synchrony between unisensory and multisensory areas was associated with faster response times (Mercier et al., 2015). In the present study, a decrease in the attentional resources for the speeded detection task may have led to disruptions in the visual stimuli resetting the phase of auditory processing and thus increasing response times for audiovisual stimuli. Similarly, Crosse et al. (2015) demonstrated that visual speech improves the cortical entrainment to auditory speech when congruent. Although this study examined ongoing audiovisual speech and not simple syllables, a similar mechanism may underlie the influence of visual syllables in our McGurk task. Thus, attention may help to promote the effect of visual speech on the cortical entrainment to auditory speech.

### Implications for Typical and Atypical Development

Both multisensory integration and attention are known to develop over the course of childhood (Brandwein et al., 2011; Coté, 2015) and are thought to play a crucial role in the development of language and reading (Ayres and Mailloux, 1981). Multisensory integration is also known to be disrupted in several developmental disorders including schizophrenia (De Jong et al., 2010; Postmes et al., 2014; Mayer et al., 2015), dyslexia (Facoetti et al., 2010; Krause, 2015), and autism (Ciesielski et al., 1995; Belmonte and Yurgelun-Todd, 2003; Foss-Feig et al., 2010; Kwakye et al., 2011; Woynaroski et al., 2013). In addition, many developmental disorders also have strong links to alterations in attentional networks, executive functioning, and higher-order processes (Cardinale et al., 2013; Kamradt et al., 2014; Sidlauskaite et al., 2016). However, very few studies have examined how the relationship between attention and multisensory integration develops or the link between multisensory integration and attention in developmental disorders and its role in shaping the trajectory, severity, and functioning of individuals with these disorders. Investigating how attention shapes multisensory integration and how this interaction develops both typically and atypically may help us to better understand the acquisition of important skillsets such as language and reading. Additionally, with an understanding of how attention and multisensory integration interact, we can develop better tools to identify developmental disorders earlier in development and use this knowledge to enhance early intervention strategies. Our findings of individual differences in the effects of attention on multisensory integration may be particularly informative for our understanding of how multisensory integration and attention may interact differently in developmental disorders and throughout development. Our findings strongly suggest that not all individuals are affected similarly by attention and may not respond in the same way to multisensory stimuli in a highly distracting environment. Additionally, the effect of attention on multisensory integration may also depend on type of multisensory information being integrated. Future studies are needed to determine how the symptomologies of various developmental disorders and complex sensory experiences such as musical training account for the individual differences observed in this study. Ultimately, our results demonstrate the importance of attention for both low-level and high-level multisensory integration. We also make evident the complexity of how attention shapes multisensory integration and how it varies between individuals. These findings have important ramifications for our understanding of both typical and atypical processing of multisensory information in our complicated and attentionally demanding environment.

## Author Contributions

LK, EA, and BE contributed to the conception and design of the project. LK, EA, BE, and KG collected the data for the project. LK, EA, BE, KG, CD, WK, and SN analyzed and interpreted the data. LK, KG, BE, WK, SN, EA, and CD wrote the paper.

## Conflict of Interest Statement

The authors declare that the research was conducted in the absence of any commercial or financial relationships that could be construed as a potential conflict of interest.
