# Supplementary figures and images for: Visual Distractors Disrupt Audiovisual Integration Regardless of Stimulus Complexity
Source: Front Integr Neurosci. 2017 Jan 20;11:1. doi: 10.3389/fnint.2017.00001 (PMC5247431; doi:10.3389/fnint.2017.00001)

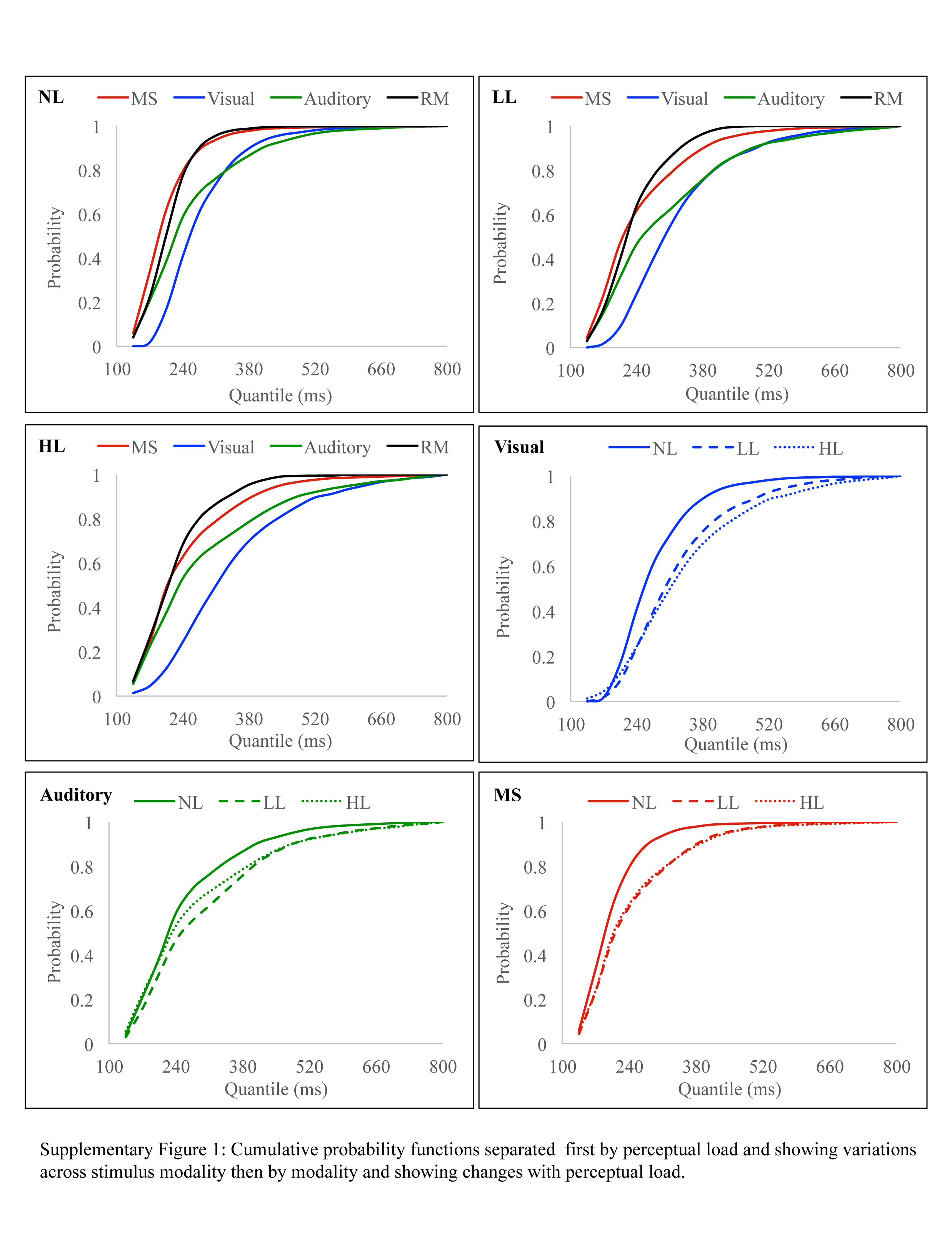

Supplement: Supplementary file 1 [file Image_1.TIF]
